# TACO: a general-purpose tool for predicting cell-type–specific transcription factor dimers

**DOI:** 10.1186/1471-2164-15-208

**Published:** 2014-03-19

**Authors:** Aleksander Jankowski, Shyam Prabhakar, Jerzy Tiuryn

**Affiliations:** 1 Computational and Systems Biology, Genome Institute of Singapore, 60 Biopolis Street, Singapore 138672, Singapore; 2 Faculty of Mathematics, Informatics and Mechanics, University of Warsaw, Banacha 2, 02-097 Warszawa, Poland

**Keywords:** Cooperativity, Dimerization, Transcription factor complexes, Dimer motifs, Chromatin accessibility, Open chromatin

## Abstract

**Background:**

Cooperative binding of transcription factor (TF) dimers to DNA is increasingly recognized as a major contributor to binding specificity. However, it is likely that the set of known TF dimers is highly incomplete, given that they were discovered using *ad hoc* approaches, or through computational analyses of limited datasets.

**Results:**

Here, we present TACO (Transcription factor Association from Complex Overrepresentation), a general-purpose standalone software tool that takes as input any genome-wide set of regulatory elements and predicts cell-type–specific TF dimers based on enrichment of motif complexes. TACO is the first tool that can accommodate motif complexes composed of overlapping motifs, a characteristic feature of many known TF dimers. Our method comprehensively outperforms existing tools when benchmarked on a reference set of 29 known dimers. We demonstrate the utility and consistency of TACO by applying it to 152 DNase-seq datasets and 94 ChIP-seq datasets.

**Conclusions:**

Based on these results, we uncover a general principle governing the structure of TF-TF-DNA ternary complexes, namely that the flexibility of the complex is correlated with, and most likely a consequence of, inter-motif spacing.

## Background

DNA-binding transcription factors (TFs) are central to the cell’s ability to recognize and decode the gene regulatory instructions contained in the genome. Their activating or repressing effect is achieved by binding to so-called motif instances, which are specific DNA sequence fragments in the regulatory regions of the genome, often in close proximity to the regulated gene. This binding was traditionally studied in isolation, despite the fact that many well-studied TFs were known to bind cooperatively to DNA by forming well-defined dimers or (in some cases) higher-order complexes. Important examples of such *direct cooperativity* include the p53 homotetramer [1], the NF-κB heterodimer [2], various bHLH dimers [3], SOX2–POU5F1 (SOX2–OCT4) dimerization in embryonic stem cells [4] and, more recently, AR–FOXA1 dimerization in prostate cancer cells [5]. In all these cases, the genomic binding sites of cooperating TFs form well-defined rigidly spaced motif complexes, i.e. motif pairs with fixed relative orientation and spacing. This is in contrast to indirect cooperativity, i.e. fuzzily spaced co-binding of any TF pairs, which can be inferred by several existing bioinformatics approaches [6-9].

**Figure 1:**
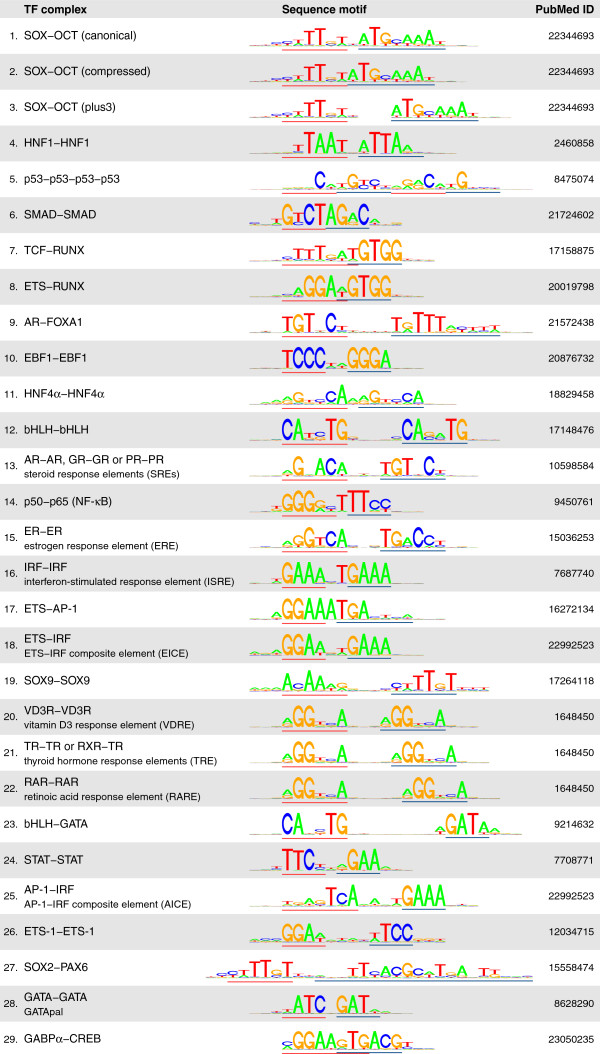
**Known dimeric DNA-binding transcription factor complexes, manually compiled from the existing biochemical literature.** For the complexes predicted in UW DNase-seq data (Figure 2B), their sequence motifs identified by TACO are shown. The remaining motifs were compiled as spacing alterations of TACO predictions or juxtaposed TRANSFAC monomers.

The list of known DNA-binding TF dimers and multimers has expanded rapidly – we recently compiled from the biochemical literature a list of 25 such complexes that have experimental support [10]. An updated and more complete list containing 29 TF complexes is shown in Figure 1. Concomitantly, numerous studies have used *in silico* analysis to computationally predict TF dimers. Since the goal of these studies was to predict specific ternary complexes of TFs with DNA, they scanned for pairs of TF-binding motifs enriched at a fixed relative orientation and spacing in regulatory regions. We previously developed one such method [10] that exploited the abundance of DNase-seq datasets available from the ENCODE consortium [11]. Others have used DNase I hypersensitivity data on a smaller scale [12], as well as TF ChIP-seq data [13,14] and also sets of promoter or enhancer regions [15,16] to define the regulatory elements of interest.

Currently, two software tools exist for performing the motif dimer enrichment analysis described above: SpaMo [13] and iTFs [12]. One important drawback of these tools is that they cannot assess enrichment of motif pairs that are so close that they overlap, even though such overlap is common [10]. We previously developed a mathematical framework for TF dimer prediction that accommodated motif overlap, and applied it to a set of DNase-seq profiles [10]. Here, we introduce TACO (Transcription factor Association from Complex Overrepresentation), a software tool that generalizes this approach. A major advance of the current work is that we have now encapsulated the algorithm into a user-configurable standalone tool. Another major improvement is that TACO is universally applicable to regulatory element annotations from any source, rather than being restricted to DNase-seq datasets. TACO also incorporates a novel motif clustering protocol (see Methods) and standardized output formats.

We applied TACO to 152 DNase-seq datasets from two sources in order to assess the consistency of the predicted dimers. We also compared TACO to SpaMo and iTFs, by benchmarking the three algorithms on the set of 29 known dimers. To demonstrate the robustness of TACO, we further applied the method to 94 ChIP-seq datasets from K562 cells.

We previously noted that TF dimers are mostly rigidly spaced and compact, and hypothesized that compactness explained rigidity [10]. Here, we use the expanded set of dimer predictions to test this hypothesis. Consistently with this hypothesis, we uncovered a significant correlation between the rigidity and compactness of predicted TF dimers.

## Results

### Consistency of DNase-seq-based TF dimer prediction

The ENCODE Project Consortium [11] provides multiple types of whole-genome open chromatin profiles, including data from DNase-seq experiments performed at the University of Washington (UW, track wgEncodeUwDnase) and Duke University (Duke, track wgEncodeOpenChromDnase). In order to obtain a comprehensive set of TF dimer predictions, and also assess the robustness and generality of our method, we ran TACO separately on both the UW and Duke collections.

**Figure 2:**
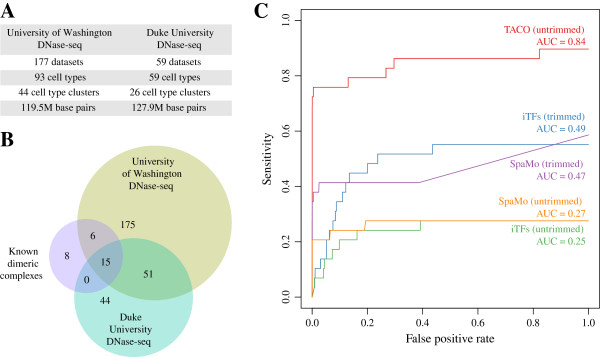
**Data sources, comparison of TF dimer predictions and dimer prediction algorithms. (A)** DNase-seq data sources. **(B)** Comparison of TF dimer predictions obtained using UW and Duke DNase-seq data. The Venn diagram illustrates the overlap between the two sets and also the set of known DNA-binding TF dimers manually compiled from the existing biochemical literature (Figure 1). **(C)** Comparison of dimer prediction algorithms. SpaMo and iTFs were evaluated both with and without motif trimming. Note that TACO does not require motif trimming. Sensitivity is shown as a function of false positive rate; Area Under Curve (AUC) is indicated.

For either of the data sources (UW or Duke), we considered all DNase-seq datasets from cell types under normal conditions (no treatment) that were not embargoed as of Jan 2013. We merged replicates and clustered cell types according to the similarity of their DNase-seq profiles, which resulted in 44 and 26 cell type clusters in UW and Duke, respectively (Figure 2A). Either of the data sources covered approximately 4% of the genome.

Application of TACO to these two sets of genomic regulatory regions yielded 247 and 110 predicted TF dimers, respectively, of which 66 were shared (Figure 2B). Note that we did not expect complete overlap, since the 93 unclustered cell types from UW and the 59 from Duke shared only 15 cell types in common. After cell type clustering, the latter 15 contributed to 14 of the 44 UW cell types and 11 of the 26 Duke cell types. We also compared predicted TF dimers with a list of 29 known TF dimers manually compiled from the existing biochemical literature (Figure 1; Additional file 1: Table S1). Note that this is an updated version of the gold-standard set used in [10]. Notably, we found that DNase-seq data from both UW and Duke were predictive of most of the known dimeric complexes.

### TACO outperforms existing dimer prediction methods

We compared TACO with the two other dimer prediction methods, SpaMo [13] and iTFs [12] using the 29 known dimers as a benchmark set of true positives (Figure 1; Additional file 1: Table S1). Henceforth, we tested 25 distinct motif pairs underlying the 29 known dimers, and as a control we included a set of 1000 random motif pairs (see Additional file 2). All the tools were applied to each of the 44 cell-type–specific UW DNase-seq datasets. Sensitivity was defined as the fraction of the 29 known dimers detected at any given *p*-value threshold. False-positive rate was defined as the fraction of the random motif dimers detected at the same threshold (Figure 2C).

SpaMo and iTFs were evaluated both with and without trimming of uninformative positions at motif edges. Motif trimming was performed as in [13] and [12]. As expected, both of these tools performed better with trimmed motifs. Notably, with motif trimming, iTFs performed marginally better than SpaMo (AUC = 0.49 vs. AUC = 0.47) despite the fact that it was not designed to predict rigidly spaced TF dimers [12]. Ultimately, TACO (AUC = 0.84) clearly outperformed the other tools; note that we did not run TACO with trimmed motifs, since TACO is able to handle motif overlap. We also found that TACO is robust to the motif sensitivity threshold chosen (Additional file 3: Figure S1). Notably, TACO and SpaMo completed the benchmarking analysis reasonably fast (2.7 and 6 hours on a single CPU machine, respectively; TACO may use multiple CPUs). However, iTFs could only complete the job in a feasible time when running on a cluster.

Comparing the three tools by applying them to the 26 cell-type–specific Duke DNase-seq datasets yielded comparable results, with TACO (AUC = 0.74) again outperforming the two other tools (Additional file 4: Figure S2A). Combining the predictions from both DNase-seq data sources gave even better performance (AUC = 0.86; Additional file 4: Figure S2B).

### Expanding the cooperativity landscape with additional DNase-seq datasets

**Figure 3:**
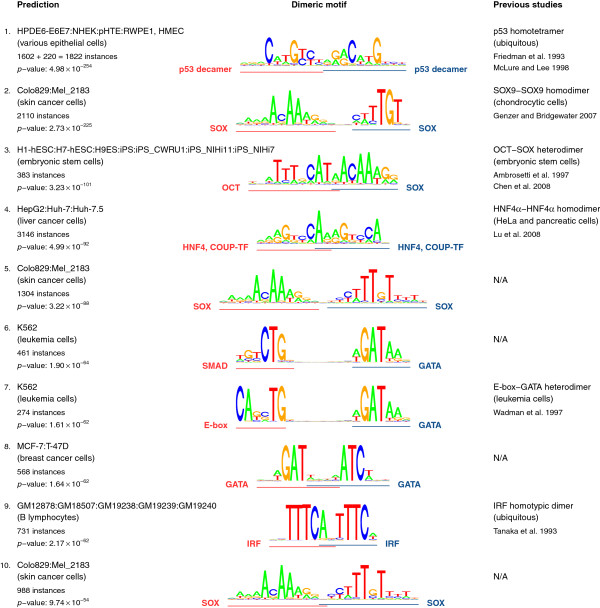
**Top 10 predicted motif dimers in Duke DNase-seq data, ranked by *p*-value.***Left column:* for each prediction, the enriched cell type, number of motif complex instances in cell-type-specific hypersensitive sites and *p*-value are indicated. *Middle column:* below each dimer motif, binding sites for individual motifs are indicated. Only the structure of the cluster seed is shown. For clarity, we have manually interpreted the motif annotations. Right column: literature citation on predicted TF dimer.

We expected that the known instances of direct TF cooperativity would tend to coincide with the most statistically significant TACO predictions, as was the case in our previous work based on UW DNase-seq data alone [10]. Focusing on the top 10 predictions derived from Duke data (Figure 3), we found 6 known interactions [1,4,17-22], the remaining 4 being novel predictions. Strikingly, while the known SOX9 homodimer [18] was detected as the 2nd ranked prediction, we also found two novel SOX homodimer motifs, ranked 5th and 10th respectively. The novel dimeric motifs are almost identical to the known SOX9 motif complex, except that the spacing between the monomer binding sites is increased or decreased by a single basepair. All three dimers were found to be specific to a cluster of melanoma (skin cancer) cell lines, consisting of Colo829 and Mel_2183. Interestingly, SOX9 is downregulated as melanocytes progress to melanoma, and its overexpression in melanoma cell lines inhibits tumorigenicity [23]. Our discovery of three distinct SOX9 homodimer binding modes in melanoma provides one candidate molecular mechanism for the biological role of this TF in melanoma formation.

Another novel prediction, GATA–SMAD dimer ranked 6th, is in line with physical and functional interaction between GATA3 and SMAD3 reported by [24]. However, we cannot rule out the alternative explanation, namely that this novel prediction is a variant of the known GATA–E-box dimer [21], ranked 7th, with only a half-site of palindromic E-box motif being bound in this case.

The final novel prediction in Figure 3, GATA–GATA, ranked 8th in Figure 3, was found specific to K562 cell line. GATA is known to be a pioneer factor [25], and has been reported to bind cooperatively to a “GATApal” palindromic composite motif: ATCWGATAAG [26]. Our predicted dimer involves a converging pair of GATA motifs, as opposed to the diverging motifs in GATApal. By extension, we therefore call this prediction “GATAcpal”.

### ChIP-seq data extend the scope of TACO

To demonstrate the ability of TACO to incorporate regulatory element annotations from multiple sources, we applied the algorithm to 127 replicates from 94 ChIP-seq experiments in K562 cells [11]. For each experiment, we downloaded from Factorbook [27] the top 5 motifs found in ChIP-seq peaks using MEME [28].

**Figure 4:**
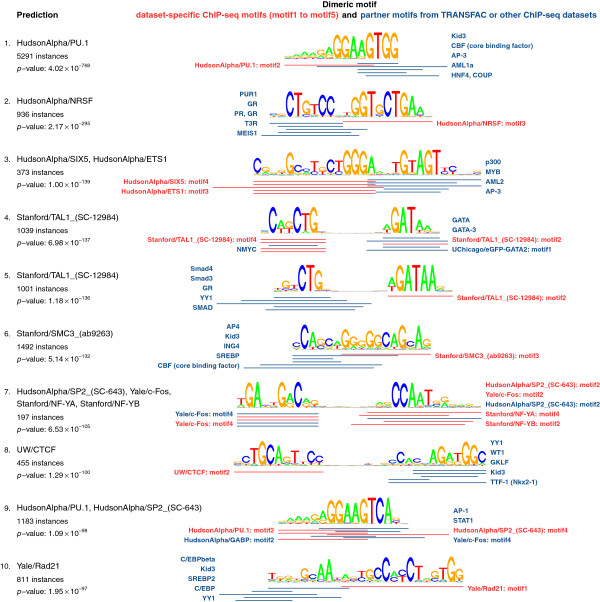
**Top 10 predicted motif dimers in K562 ChIP-seq peaks, ranked by *p*-value.***Left column:* for each prediction, the names of enriched ChIP-seq datasets, followed by the number of motif complex instances and *p*-value in most significantly enriched dataset. *Right column:* below each dimer motif, the locations and names of underlying individual motifs are indicated for the top 5 overrepresented motif complexes. Red motifs correspond to the TF immunoprecipitated in an enriched ChIP-seq dataset, whereas blue motifs originate from TRANSFAC or other ChIP-seq datasets. For clarity, the red lines were drawn only once if the corresponding motif was shared across all 5 complexes.

We used TACO to scan for motif complexes that contained at least one of the 5 motifs discovered in the respective dataset. The partner motif in the complex could be from the TRANSFAC database or from the entire set of motifs discovered in all K562 datasets. In total, our analysis yielded 81 predicted TF dimers, of which the top 10 are shown in Figure 4. Ranked 1st is the known ETS–RUNX dimer [14], which was found in ChIP-seq peaks for PU.1, a transcription factor from the ETS family.

The 2nd ranked prediction, found in ChIP-seq peaks for NRSF (REST), actually represents a full-length, monomeric REST motif [29]. It was predicted by TACO as a dimeric motif complex because “HudsonAlpha/NRSF: motif3”, the third-ranked motif discovered by MEME within REST ChIP-seq peaks, is actually only a fragment of the full-length REST motif, and the remaining fragment is very similar to the motif for nuclear receptors such as GR and PR.

The 4th ranked prediction is the known GATA–E-box motif complex [21], which was also identified in the above-described analysis of Duke DNase-seq datasets (ranked 7th in Figure 3). Here, it is overrepresented in ChIP-seq peaks for the E-box-binding factor TAL1. Not surprisingly, among the top 5 motifs found in these ChIP-seq peaks, there is an E-box motif “Stanford/TAL1_(SC-12984): motif4”. The top 5 motifs also include the GATA motif “Stanford/TAL1_(SC-12984): motif2”. Such secondary TF motifs have been frequently reported in addition to the canonical ones [27]. However, the biophysical interpretation of such secondary motifs is usually unclear. They could be a consequence of tethered binding, functional cooperativity or actual dimerization. These diverse mechanistic explanations can be distinguished more easily with the help of TACO spacing analysis. In this case, it is clear that the secondary GATA motif at TAL1 ChIP-seq peaks is a consequence of GATA–TAL1 heterodimerization on DNA.

### Dynamic landscape reveals low TF dimer reuse across cell types

**Figure 5:**
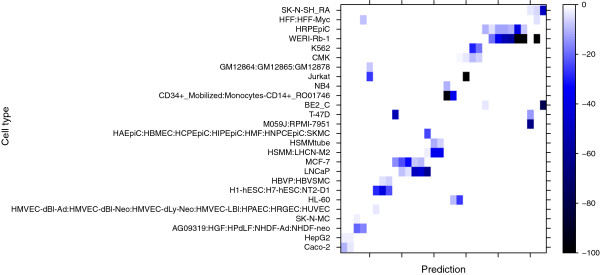
**Dynamic landscape of predicted TF dimers across cell types.** Each column of the heatmap represents a motif dimer predicted in UW DNase-seq data in more than one cell type. Dimers predicted only in a single cell type are not shown. Color intensity indicates the motif complex enrichment *p*-value in the given cell type. Rows and columns were clustered using complete linkage method with binary metric.

The vast majority of TF dimers predicted in DNase-seq data were found specific to a single cell type only (87% or 215/247 in UW, 89% or 98/110 in Duke). Out of the 32 remaining dimers in UW, 29 were predicted in exactly two cell types (Figure 5) and usually found to be reused between related cell types (e.g. prostate cancer LNCaP and breast cancer MCF-7). Note that these predictions originated from disjoint sets of genomic regions (i.e. cell-type–specific hypersensitive sites), so the predictions in different cell types are independent. A similar trend of low TF dimer reuse was observed in Duke DNase-seq data (Additional file 5: Figure S3).

### Association between rigidity and compactness of TF dimers

**Figure 6:**
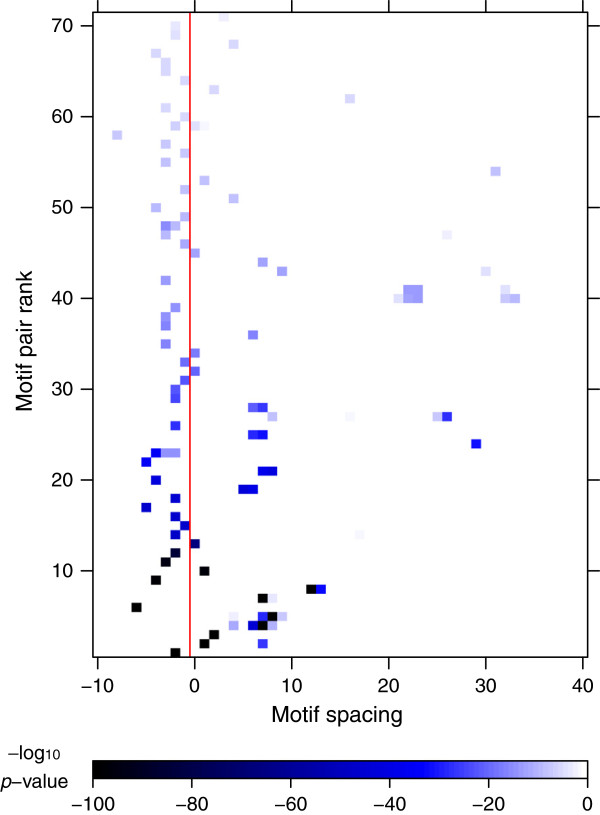
**Wide range of motif spacings for TF dimers predicted in K562 cells.** Predicted dimers that varied only in their spacing (same motif pair and orientation) were grouped together and ranked by the *p*-value of the most significant spacing. For each such group of dimer predictions in K562 ChIP-seq peaks, we show the motif complex enrichment *p*-value as a function of motif spacing. Spacings to the left of the red line correspond to overlapping motifs.

Notably, the analysis of overrepresented motif complexes in ChIP-seq peaks yielded multiple long-range interactions (spacing >15 bp), which were not discovered in our previous analyses of DNase-seq data (Figure 6). Most dramatically, we observed that in two such cases, ranked 40th and 41st, up to 5 motif spacings were significantly overrepresented. Both of these predictions involved NF-Y homodimers, as did yet another of the predictions (Additional file 6: Figure S4A). Of the 9 predicted NF-Y homodimers, 5 were direct repeats, 3 were divergent palindromes and 1 was a convergent palindrome. The 5 different spacings for the NF-Y direct repeat were broken up into two clusters one turn apart, and therefore phased to be on the same side of the DNA double helix. Another relatively widely spaced (>5 bp) interaction mentioned earlier, GATA–E-box, similarly permitted flexible spacing (Additional file 6: Figure S4B).

**Figure 7:**
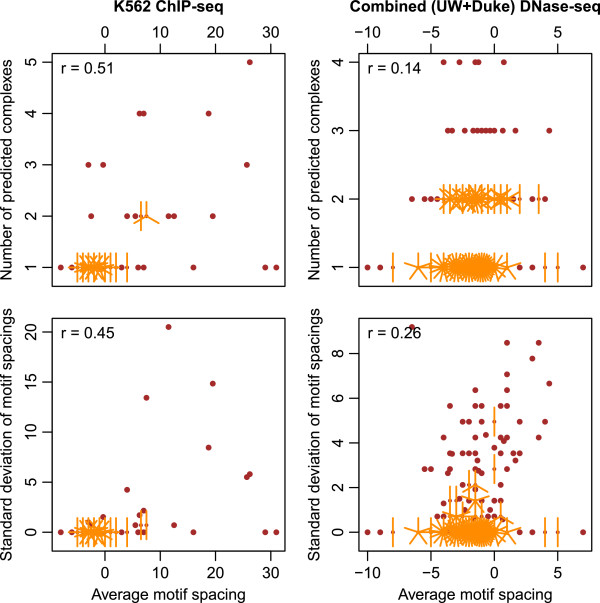
**Positive association between average motif spacing and flexibility of motif dimers.***Left column:* predictions in K562 ChIP-seq peaks, *right column*: combined predictions from UW and Duke DNase-seq data. *Upper row:* sunflower plots show the number of predicted motif spacings for a group of dimer predictions as a function of the average of their motif spacings. In case of data points occurring more than once, their count is indicated by the number of petals (orange lines). *Lower row:* sunflower plots show the standard deviation of predicted motif spacings as a function of average motif spacing. The Pearson correlation coefficients are shown for all plots.

In order to quantify a potential association between rigidity and compactness of TF dimers, we aggregated TACO predictions derived from K562 ChIP-seq data into groups that varied only in their motif spacing (see Methods), as in Figure 6. We then found Pearson correlation coefficient of *r* = 0.51 between the number of enriched complexes for a motif pair and their average motif spacing (Figure 7, upper left). The difference in average motif spacing calculated within the prediction groups, compared between completely rigid motif complexes (single-spacing) and flexible complexes (more than one spacing) was found highly significant (*p* = 4.07e-06, Mann–Whitney U test). Thus, we see a highly significant correlation between the rigidity and compactness of predicted TF dimers.

In order to test the generality of the abovementioned correlation, we applied the same approach to the combined set of DNase-seq dimer predictions, obtained using UW or Duke data. Again, we observed a positive Pearson correlation of *r* = 0.53 between the number of predicted complexes for a motif pair and their spacing. However, we noticed that four of the complexes in this case dominated the correlation coefficient by virtue of having outlier values for the motif spacing; their motif spacing was more than 5 interquartile ranges above the third quartile. When these four data points were discarded, the correlation coefficient dropped to *r* = 0.14 (Figure 7, upper right). However, we still observed significantly larger average motif spacing among flexible complexes as compared to the completely rigid complexes (*p* = 0.014).

We further tested whether a more quantitative measure of dimer flexibility would also support the above findings on the structural properties of TF dimers. Consistently, we found that the average motif spacing also correlates with the standard deviation of motif spacings for a motif pair (Figure 7, lower left and right). In this case, the Pearson correlation coefficients were *r* = 0.45 for K562 ChIP-seq dimers and *r* = 0.47 for combined DNase-seq dimers (*r* = 0.26 after outlier removal). In summary, we found that the rigidity and compactness of motif complexes are consistently correlated, by multiple measures in two different data types.

## Discussion

Typically, TFs bind to only a very small fraction of their motif matches in the vast human genome. It is thought that the remaining motif matches remain unbound because they lie in closed chromatin [11]. This model should not apply to pioneer factors, since they have the ability to bind closed chromatin. It is therefore not clear how do pioneer factors achieve binding specificity. We previously discovered multiple potential homo- and heterodimeric complexes involving FOXA1, and hypothesized that this pioneer factor could achieve binding specificity by exploiting a multiplicity of dimeric binding modes [10]. The pioneer factor GATA may constitute yet another example of this phenomenon, given its multiple known and newly predicted dimeric binding modes (GATA–E-box, GATApal, GATAcpal).

We have so far assumed that the existence of a preferred motif spacing for a TF pair is indicative of dimeric binding. However, there is one other possible explanation that must be kept in mind. It has been shown that Smad4 dimers can bind cooperatively to DNA even in the absence of direct physical contacts [30]. The authors of this study suggested that DNA conformational changes induced by TF binding could be a mechanism for cooperative binding of specific Smad4 homo- and heterodimers. It is conceivable that some of our predicted TF pairs might cooperate via allosteric changes in DNA structure rather than direct protein-protein contacts.

We previously showed that TF dimers were both rigid and compact, and hypothesized based on qualitative structural arguments that their rigidity was a consequence of their compactness [10]. Such a causal relationship could arise for two reasons. Firstly, TF pairs binding widely spaced motifs are likely to form protein-protein contacts via their DNA-distal domains, or even via intervening cofactors. Such a configuration would in general be more flexible than direct physical contact between the DNA-binding domains. Secondly, a widely spaced complex might also gain flexibility from the greater deformability of the long stretch of intervening DNA. The widely spaced complexes found in K562 cells provided us with an opportunity to test the above hypothesis. Our results indicate that TF dimers that bind widely spaced motif pairs are significantly more flexible in their spacing, thus providing statistical support for a causal relationship between compactness and rigidity (Figure 7). While our analysis provides the first evidence, further biochemical experiments are required to explore this relationship in greater detail.

In cases of very high inter-domain flexibility, as is perhaps true of NF-Y, even the relative orientation of individual motifs may vary. The NF-Y complex contains three proteins, NF-YA, NF-YB and NF-YC, of which only NF-YA forms specific contacts with DNA [16]. Thus, the NF-Y “dimer” motifs we identified are likely to be bound by pairs of such trimers, i.e. hexamers. It is possible that inter-trimer contacts are mediated not by the DNA-binding NF-YA subunit, but by the DNA-distal NF-YB or NF-YC subunits. Interestingly, the NF-Y motif was recently reported to form well-defined complexes of fixed spacing with E-box, E2F and TATA-box motifs at promoters genome-wide [16], suggesting that the ternary complexes identified here are not the only cooperative interactions involving NF-Y. The same study also showed that NF-Y was unusually adept at binding genomic regions that showed no activating or repressive histone marks, suggesting that the TF acts as a pioneer factor. This is again consistent with our previous hypothesis that pioneer factors derive their DNA binding specificity from multiple dimeric binding modes.

Although the TF dimers predicted by TACO are generally rigidly spaced, it is conceivable that this reflects to some extent an ascertainment bias of the algorithm. Dimers with highly flexible spacing would be harder to detect by this method, if they resulted in only weak enrichment of motif pairs at any given spacing. Similarly, the fact that all of the 29 known TF dimers we extracted from the literature are rigid or semi-rigid could also be questioned; one could hypothesize that existing biochemical assays for detecting cooperative dimerization on DNA are somehow biased against flexibly spaced dimers. However, we are not aware of any experimentally validated instances of TF dimers that can bind *cooperatively* with highly flexible motif spacing. Notably, in a recent study, even though the algorithm used to predict TF dimers permitted some flexibility in the spacing, all of the experimentally validated dimers turned out to be rigid, i.e. they bound with high affinity only at a single motif spacing [12]. Thus, the evidence so far is strongly weighted towards rigid or semi-rigid TF dimers.

## Conclusions

We have demonstrated the generality and consistency of TF dimer predictions made by TACO by applying the algorithm to 152 DNase-seq datasets and 94 ChIP-seq datasets from the ENCODE Project. Moreover, we showed that TACO clearly outperforms existing dimer prediction tools when benchmarked on the set of 29 known dimers. Based on all TACO predictions, we found that TF dimers that bind widely spaced motif pairs are significantly more flexible in their spacing. Overall, we expect TACO to be widely applicable, since thousands of regulatory element datasets will be available in the near future. We also anticipate its application to regulatory annotations from assay types other than those discussed here, since the algorithm allows a great deal of flexibility in data type and mode of analysis.

## Methods

### Overview of the method

Our approach builds on the comprehensive model of motif co-occurrence constructed in [10]. The method is based on analysis of motif complex enrichment within regulatory regions specific to individual cell types. To detect overrepresentation, we compare the occurrence frequency of a TF complex in the target dataset (cell-type–specific open chromatin regions, for example) to the frequency of the same complex in the union of all input datasets across all cell types.

Given a motif complex, i.e. a specific orientation and spatial arrangement of two motifs, we define motif spacing as the number of intervening base pairs between the proximal edges of the two contributing motifs (negative values indicate motif overlap). By default, all the possible motif complexes within 50 bp spacing are screened for enrichment, in each target dataset separately. The *p*-values are calculated from Bernoulli schema and Bonferroni-corrected.

### Identification of dataset-specific predictions

We use DNA sequence motifs as models of TF binding specificity. In the default setting, we consider all possible pairs of the motifs provided. For each pair of motifs we test all possible compact motif complexes (all relative orientations and, by default, motif spacing of at most 50 bp) for enrichment in each of the target datasets. It should be noted that TACO can seamlessly handle the statistics of overlapping motif pairs, a property not shared by existing algorithms. This is an important feature, since a sizeable fraction of known TF dimers bind overlapping motif pairs [10].

To quantify enrichment, we count the number of motif complex instances in each target dataset, and compared it against the number of instances in the background model. The background model is based on the control dataset, defined as the union of all regulatory regions from all cell types. The enrichment is calculated taking into account the difference in motif co-occurrence frequency between foreground (target) and background (control) datasets [10].

Motif databases very often contain multiple motifs for the same TF, or very similar motifs for different TFs. For this reason, a single underlying TF-TF interaction often results in the detection of multiple, highly similar motif complexes by TACO. We therefore cluster the overrepresented motif complexes, taking into account their similarity (measured by Euclidean distance) and overlap of their genomic instances, as described below.

### Clustering of overrepresented motif complexes

We rank the overrepresented motif complexes by *p*-value in ascending order (i.e. starting from the most highly enriched complex). Let us denote them by *R*_*1*_*, …, R*_*N*_. In order to cluster the complex *R*_*n*_, we loop through *k = 1, …, n–1* and iteratively check if *R*_*n*_ is similar to *R*_*k*_, as described below. If any of the comparisons yields a positive result, we immediately merge *R*_*n*_ into the cluster containing *R*_*k*_. If the complex *R*_*n*_ cannot be incorporated into any of the existing clusters, a new cluster is created, with *R*_*n*_ as the *cluster seed*. In particular, the most enriched overrepresented motif complex, i.e. *R*_*1*_, gives rise to the first cluster.

To compare *R*_*n*_ to *R*_*k*_, the following three tests are performed. If any of the three tests results in a positive outcome, the two complexes are deemed to be similar.

#### Test 1: motif complex identity

The first test is attempted only if *R*_*k*_ is the cluster seed of a previously established cluster. If *R*_*n*_ and *R*_*k*_ share the same motif complex, then *R*_*n*_ is *joined by motif complex identity* to the cluster of *R*_*k*_. It occurs when the same motif complex is found overrepresented in different target datasets.

#### Test 2: dimer motif similarity

The second test is attempted only if *R*_*k*_ is a *signature motif complex*, i.e. the cluster seed or joined by motif complex identity to its cluster. Let ED^2^(*R*_*n*_, *R*_*k*_) be the squared Euclidean distance between the dimer motifs for complexes *R*_*n*_ and *R*_*k*_. The simplest motif similarity criterion would be to impose a threshold on ED^2^. However, our approach allows highly specific motifs (those with high information content) to be further apart in Euclidean space, and still be considered similar. We therefore employ a distance threshold that is an affine function of the information content. If ED^2^(*R*_*n*_, *R*_*k*_) < *α* ∙ IC(*R*_*k*_) + *β*, where *α* and *β* are user-provided parameters, and IC(*R*_*k*_) is the information content of the dimer motif for *R*_*k*_, then *R*_*n*_ is *joined by dimer motif similarity* to the cluster of *R*_*k*_.

#### Test 3: overlap of genomic instances

The third test is attempted only if *R*_*k*_ is a signature motif complex or joined by dimer motif similarity. Let *C*_*12*_(*R*_*n*_∩*R*_*k*_) be the number of their overlapping genomic instances (note that only overlaps conforming to the most common relative spatial arrangement of *R*_*n*_ and *R*_*k*_ are counted). Intuitively, we would like to capture the number of excess instances of *R*_*n*_ that are not also instances of *R*_*k*_.

As described in detail in [10], the enrichment *p*-value of *R*_*n*_ is calculated as the probability of observing at least *C*_*12*_(*R*_*n*_) successes in *N*_*12*_(*R*_*n*_) trials of the Bernoulli process with probability of success *f*_*12*_*∙* (*b*_*12*_(*R*_*n*_)/*b*_*12*_), where *C*_*12*_(*R*_*n*_) is the actual number of *R*_*n*_ instances in the target dataset, *N*_*12*_(*R*_*n*_) is the number of all its possible occurrences in the target dataset, *b*_*12*_(*R*_*n*_) is the probability of observing *R*_*n*_ in the control dataset, and *f*_*12*_ and *b*_*12*_ are the probabilities of observing the pair of motifs constituting *R*_*n*_ within a reasonable range of structures in the target and control dataset, respectively. The success probability of this Bernoulli process combines two components: the “base” probability *b*_*12*_(*R*_*n*_) of observing the motif complex *R*_*n*_ in the control dataset, and the factor *f*_*12*_/*b*_*12*_ accounting for the enrichment of the underlying motif pair (i.e. motif complexes regardless of their spacing) in the target dataset.

Now we introduce *E*_*12*_(*R*_*n*_) = *N*_*12*_(*R*_*n*_) ∙ *f*_*12*_ ∙ (*b*_*12*_(*R*_*n*_)/*b*_*12*_) as the expected number of instances of *R*_*n*_ following from the null model. Consequently, the number of excess instances over the null model now amounts to *C*_*12*_(*R*_*n*_)–*E*_*12*_(*R*_*n*_). If *C*_*12*_(*R*_*n*_∩*R*_*k*_) ≥ *γ* ∙ (*C*_*12*_(*R*_*n*_)–*E*_*12*_(*R*_*n*_)), where *γ* is a user-provided parameter, then *R*_*n*_ is *joined by overlap of genomic instances* to the cluster of *R*_*k*_.

### Implementation and applicability

TACO is a standalone C++ software tool. Its mandatory inputs are: reference genome sequence (FASTA format) and a list of TF motifs or a motif database. Accepted motif formats include TRANSFAC [31], JASPAR [32], SwissRegulon [33] and MEME [28] output. Moreover, a collection of genome-wide sets of regulatory regions should be provided (BED format). TACO can handle input regulatory region datasets of two kinds: strongly cell-type–specific or weakly cell-type–specific. Each input dataset should be declared as strongly or weakly specific (these two kinds can be provided simultaneously). In our previous work [10], and also in this study, DNase-seq datasets were processed according to the strongly specific paradigm. In contrast, ChIP-seq datasets considered here were treated as weakly specific.

Strongly and weakly cell-type–specific datasets are translated using different approaches into target datasets for TF dimer prediction. Regulatory regions of strongly specific datasets are intersected with each other, and only the non-overlapping (unique) portions are retained as target regions. In contrast, the weakly specific datasets are directly used as target datasets, without modification. The union of all input regulatory regions is used as a control dataset in order to build the null model of motif complex occurrence.

The open chromatin datasets which could be used include publicly available DNase-seq data from the ENCODE Project [11]. The input datasets can be provided as multiple replicates per cell type, to be merged by TACO within each cell type. In this way, closely related cell types, e.g. with similar genome-wide DNase I hypersensitivity profiles, may be merged as well.

The scope of the analysis may be narrowed down by screening for enrichment only in a subset of the target datasets. Moreover, instead of scanning for enrichment of all possible motif pairs, one or both of the motifs forming the motif complex can be fixed by the user. Below we provide three typical use cases for TACO.

#### Prediction of overrepresented motif complexes in a collection of DNase-seq datasets

All possible motif complexes are screened for enrichment in all cell-type-specific open chromatin regions. As stated, such analysis follows the concept of [10]. Alternatively, only some of the datasets could be screened, with the remaining open chromatin datasets contributing only to the control set.

#### Prediction of overrepresented motif complexes in ChIP-seq peaks

The motifs of immunoprecipitated TFs are supplied, and all motif complexes with all possible partner motifs from the database are screened for enrichment in ChIP-seq peaks. This approach has previously been used by [13]. The collection of ChIP-seq peaks should be large enough to provide a representative control set. For example, all publicly available ChIP-seq datasets from the ENCODE Project for a given cell type could be used.

#### Analysis of cooperative interactions between a given pair of TFs with known motifs

Some TF dimers allow for multiple spacings, and are overrepresented only in certain datasets (see Results). Given a pair of motifs of interest, all possible motif complexes are screened for enrichment in all datasets.

#### Execution time and output

One of our priorities while developing TACO was to make the analyses computationally tractable. Comprehensive analyses using two sources of DNase-seq data, described in the Results section above, where we took as input 964 vertebrate TF affinity motifs from TRANSFAC Professional [31], requires the testing of 2.57 billion hypotheses. TACO completes this task in approximately 6 hours, using 16 cores of a 3.33 GHz machine and up to 11 GB of memory.

As output, TACO provides a multidimensional view of overrepresented cell-type–specific motif complexes. First, TACO clusters the enriched motif complexes as described above, and treats each cluster as a single predicted TF dimer. For each overrepresented motif complex within a cluster, the locations of all its genomic occurrences are reported. We also provide the position weight matrices inferred by counting nucleotide frequencies at each position within its genomic instances. Moreover, TACO also provides statistics that can be used to visualize the distribution of enrichment *p*-values using a Q-Q plot, and to generate spacing plots as in Figure 6.

The source code for TACO is freely available under the GNU GPL license, along with examples and documentation, at http://bioputer.mimuw.edu.pl/taco/.

### Analysis of motif spacing flexibility

We defined motif spacing to be the number of intervening nucleotides between the proximal basepairs of the two motifs. In order to make the definition robust, we calculated motif spacing on the basis of trimmed motifs. Motif trimming was implemented as in [13], by eliminating flanking columns with information content less or equal 0.25 bit from both sides of the individual motifs. Note that motif trimming was only used to calculate motif spacing; TACO did not require motif trimming.

To characterize the flexibility of TF-TF-DNA complexes, we grouped together TACO predictions that could have arisen from multiple spacings of the same TF dimer. In other words, we grouped together predicted motif complexes that shared the same pair of motifs in the same orientation, and varied only in their motif spacing. In the case of DNase-seq data, we only grouped predictions arising from the same dataset (for example, UW DNase-seq in GM12878 cells). Note that motif complexes within a group were constrained to all have the same left-right ordering of the individual motifs.

## Availability and requirements

**Project name:** TACO (Transcription factor Association from Complex Overrepresentation)

**Project home page:**http://bioputer.mimuw.edu.pl/taco/

**Operating system(s):** Unix-like, such as Linux and Mac OS X

**Programming language:** C++

**Other requirements:** R or standalone R math library

**License:** GNU GPL

**Any restrictions to use by non-academics:** None

## Competing interests

The authors declare that they have no competing interests.

## Authors’ contributions

AJ wrote the software and processed the data. AJ, SP and JT designed the experiments, analyzed the data and wrote the manuscript. All authors read and approved the final manuscript.

## Supplementary Material

Additional file 1: Table S1 Known dimeric DNA-binding transcription factor complexes, in a machine-friendly format. Known dimeric DNA-binding transcription factor complexes, manually compiled from the existing biochemical literature, represented as TRANSFAC motif complexes. Click here for file

Additional file 2 Details on benchmarking the dimer prediction tools. Click here for file

Additional file 3: Figure S1 Robustness of TACO with respect to motif sensitivity threshold chosen. Area Under Curve (AUC) calculated as in Figure 2C in the main text. Red dotted line indicates the 0.8 sensitivity threshold used throughout this study. Click here for file

Additional file 4: Figure S2 Comparison of dimer prediction algorithms. As in Figure 2C, with algorithms evaluated using (A) Duke and (B) combined (UW + Duke) DNase-seq data. Click here for file

Additional file 5: Figure S3 Dynamic landscape of predicted TF dimers across cell types. As in Figure 5, but for motif dimers predicted in Duke DNase-seq data. Click here for file

Additional file 6: Figure S4 Predicted long range motif dimers in K562 ChIP-seq data. As in Figure 4, (A) NF-Y homotypic dimers and (B) GATA–E-box heterodimers predicted in K562 ChIP-seq data are shown in detail. Click here for file
