# Supplementary material for: TACO: a general-purpose tool for predicting cell-type–specific transcription factor dimers
Source: BMC Genomics. 2014 Mar 19;15:208. doi: 10.1186/1471-2164-15-208 (PMC4004051; doi:10.1186/1471-2164-15-208)
Supplement: Additional file 2 — Details on benchmarking the dimer prediction tools. [file 1471-2164-15-208-S2.pdf]

TACO: a general-purpose tool for predicting cell-type–specific transcription factor dimers

Aleksander Jankowski, Shyam Prabhakar and Jerzy Tiuryn

ADDITIONAL METHODS

Benchmarking the dimer prediction tools

We compared TACO with the two other dimer prediction methods, SpaMo (13) and iTFs (12), by benchmarking the the three algorithms on the set of 29 known TF dimers manually compiled from the existing biochemical literature (Table 1; Supplementary Table 1).

Since the known dimers were used as a set of true positives, we tested 25 distinct motif pairs underlying the 29 known dimers. As a control, we added a set of 1000 random motif pairs, which were randomly chosen from all motif pairs which could be possibly formed using all 964 vertebrate motifs from TRANSFAC Professional 2011.2 (17). We also ensured that the set of 1000 random motif pairs does not overlap with the set of 25 positive motif pairs.

Each of the tools (TACO, SpaMo and iTFs) was applied to each of the 44 cell-type–specific DNase-seq datasets from University of Washingon (UW) and each of the 26 cell-type–specific DNase-seq datasets from Duke University (Duke). In these datasets, we masked repetitive regions (as identified by RepeatMasker and Tandem Repeat Finder) and coding regions (extracted from Ensembl). Options specific to each of the tools are reported in the next subsections.

SpaMo and iTFs were evaluated both with and without trimming of uninformative positions at motif edges. Motif trimming was implemented externally and performed as in (13) and (12), by eliminating flanking columns with information content less or equal 0.25 bit from both sides of the individual motifs. Note that we did not run TACO with trimmed motifs, since TACO is able to handle motif overlap.

Each of the tools was applied to each cell-type–specific dataset separately to calculate enrichment *p*-values for all motif complexes which could be formed from the abovementioned motif pairs. Note that iTFs uses binned spacing, so the *p*-values were provided for each spacing interval within each mutual motif orientation. Since we do not have complete *a priori* information on the cell-type–specificity of known dimers, we combined all the enrichment *p*-values across datasets by choosing the most significant *p*-value for a given motif complex. In the case of combined (UW+Duke) study, the enrichment *p*-values were combined across data sources as well.

Sensitivity was defined as the fraction of the 29 known dimers (i.e. known motif complexes) detected at any given *p*-value threshold. False-positive rate was defined as the fraction of the random motif dimers (i.e. all the other motif complexes) detected at the same threshold.

Benchmarking parameters for TACO

TACO was run with default options (in particular, MaxMotifSpacing = 50). Individual motif matches were identified using the recommended criterion for TRANSFAC, i.e. Sensitivity = 0.8. In addition, to ensure that all *p*-values are reported, we specified TargetInstancesThreshold = 0, FoldChangeThreshold = 0.0 and PValueThreshold = Inf.

We also minimized the motif complex clustering, which was irrelevant to the benchmarking, in order to adequately compare the execution time. Hence, we specified ClusteringDistanceConstant = 0.0, ClusteringDistanceMultiplier = 0.0 and ClusteringOverlapThreshold = Inf.

Benchmarking parameters for SpaMo

SpaMo was run using "spamo -trim 0 -cutoff 1 -margin 50 -keepprimary -bgfile *[background file] [dataset] [motif1]*.meme *[motif2]*.meme". The background file, containing the nucleotide frequencies, was generated by fasta-get-markov from the union set of all cell-type–specific DNase-seq datasets considered.

Benchmarking parameters for iTFs

iTFs was run with distance ranges as specified in [12], namely 0-10, 10-25, 25-50 and 50-100 bp.

We noted that due to the spacing binning, three known hormone receptor homodimers (rows 20-22 in Table 1) cannot be easily distinguished. These motif complexes share the same motifs and orientation, and differ only by their spacing, which falls into the same distance range. To resolve this ambiguity, we referred to TACO and SpaMo predictions and found that these complexes were identified as enriched in different datasets.

In the case of UW data, TR−TR or RXR−TR (row 21) was most overrepresented in WERI-Rb-1 cell type (uncorrected TACO *p*-value = 1.53e-32), RAR−RAR (row 22) in SK-N-SH_RA (p = 2.65e-4) and VD3R−VD3R (row 20) in WERI-Rb-1 (p = 0.039) and NB4 (p = 0.058). Consequently, for iTFs analysis of the single motif pair yielding these complexes in UW data, we separated WERI-Rb-1, SK-N-SH_RA and NB4 datasets, considering them as indicators of three different dimers, and excluded all the other datasets.

In the case of Duke data, we found no significant cell-type–specific overrepresentation of the motif complexes discussed above. However, to make the results comparable, we referred to the smallest (albeit insignificant) TACO *p*-values and found that TR−TR or RXR−TR was most overrepresented in HMEC cell type, RAR−RAR in LNCaP and VD3R−VD3R in 8988T. Consequently, for iTFs analysis of the single motif pair yielding these complexes in Duke data, we separated HMEC, LNCaP and 8988T datasets as above, and excluded all the other datasets.

In the case of combined (UW+Duke) study, for iTFs analysis of the single motif pair discussed above, we separated the three datasets as in the UW case, and excluded all the other datasets, including all Duke ones.

For all the other motif pairs, we combined all the datasets (cell types) as previously described.
