# Supplementary figures and images for: TACO: a general-purpose tool for predicting cell-type–specific transcription factor dimers
Source: BMC Genomics. 2014 Mar 19;15:208. doi: 10.1186/1471-2164-15-208 (PMC4004051; doi:10.1186/1471-2164-15-208)

AUC of fraction of known dimers recovered, as a function of FDR

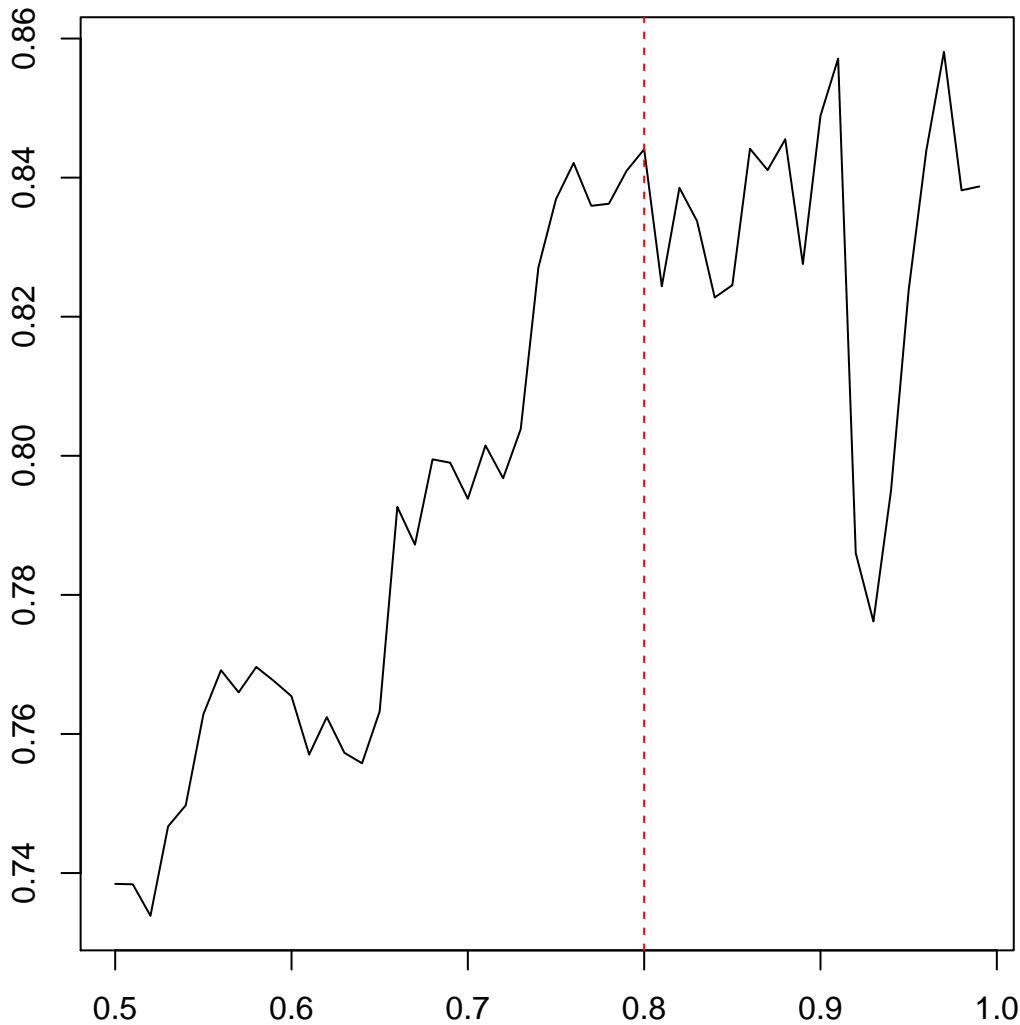

Motif sensitivity threshold

Supplement: Additional file 3: Figure S1 — Robustness of TACO with respect to motif sensitivity threshold chosen. Area Under Curve (AUC) calculated as in Figure 2C in the main text. Red dotted line indicates the 0.8 sensitivity threshold used throughout this study. [file 1471-2164-15-208-S3.pdf]

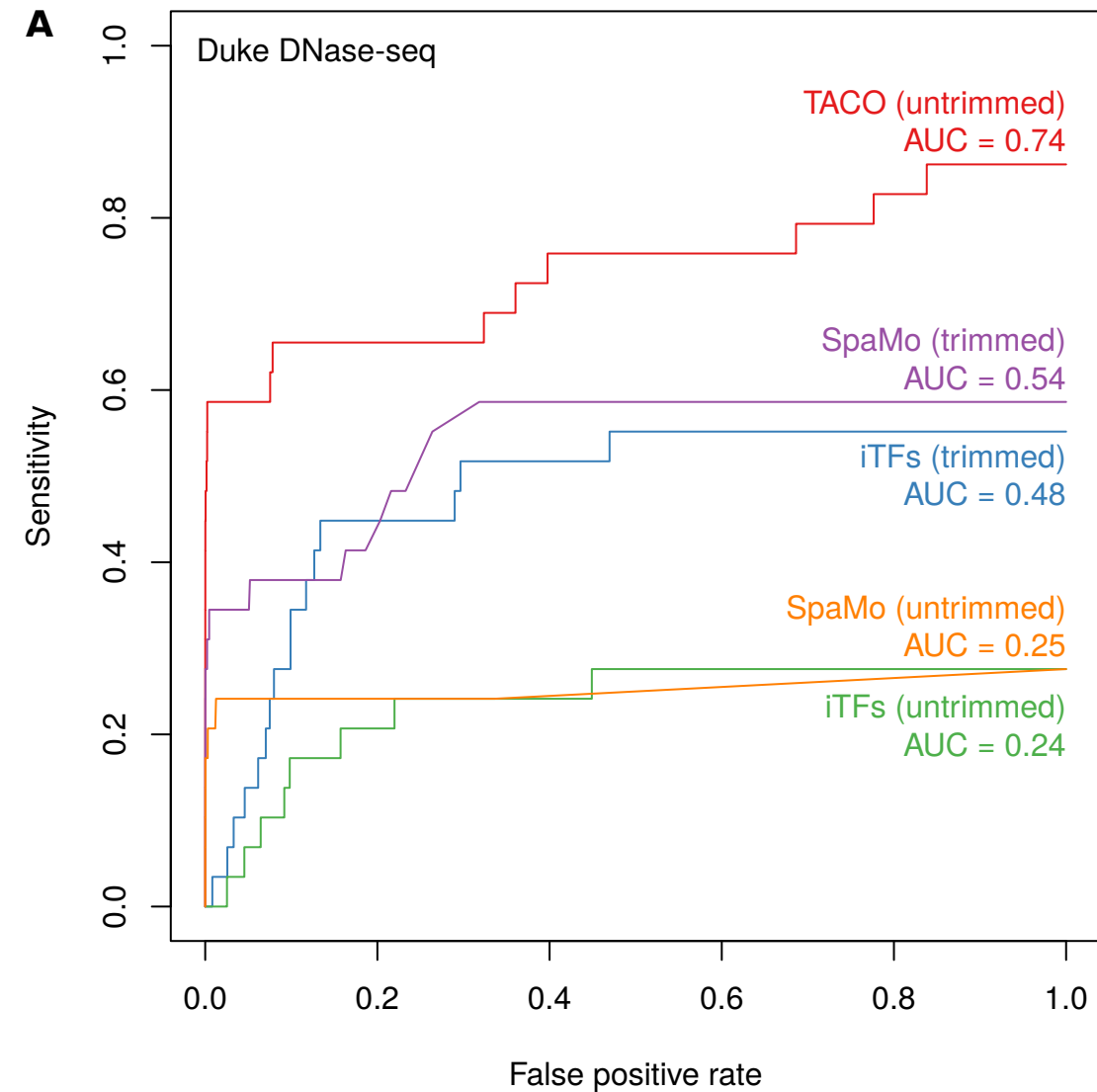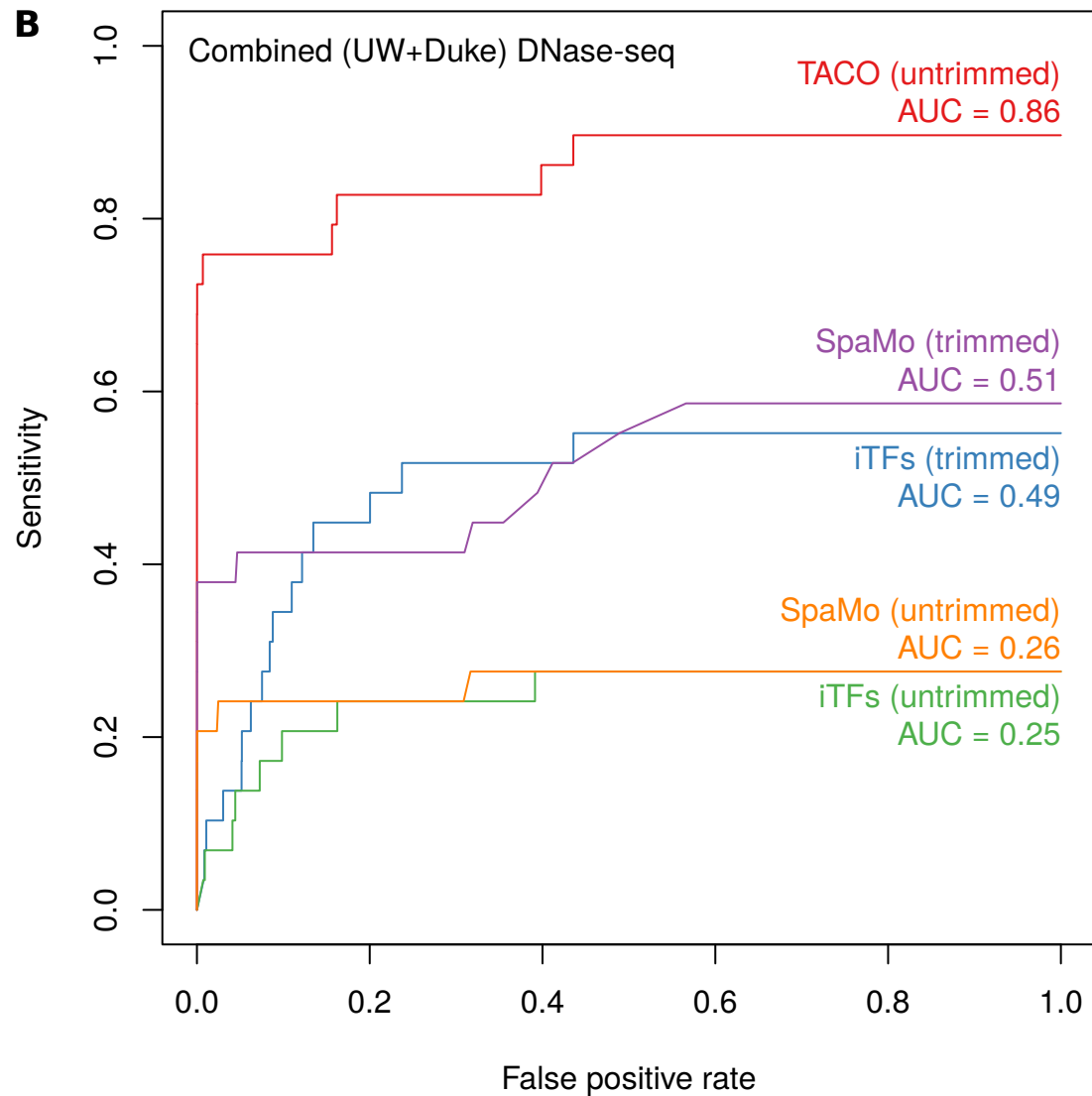

Supplement: Additional file 4: Figure S2 — Comparison of dimer prediction algorithms. As in Figure 2C, with algorithms evaluated using (A) Duke and (B) combined (UW + Duke) DNase-seq data. [file 1471-2164-15-208-S4.pdf]

Cell type

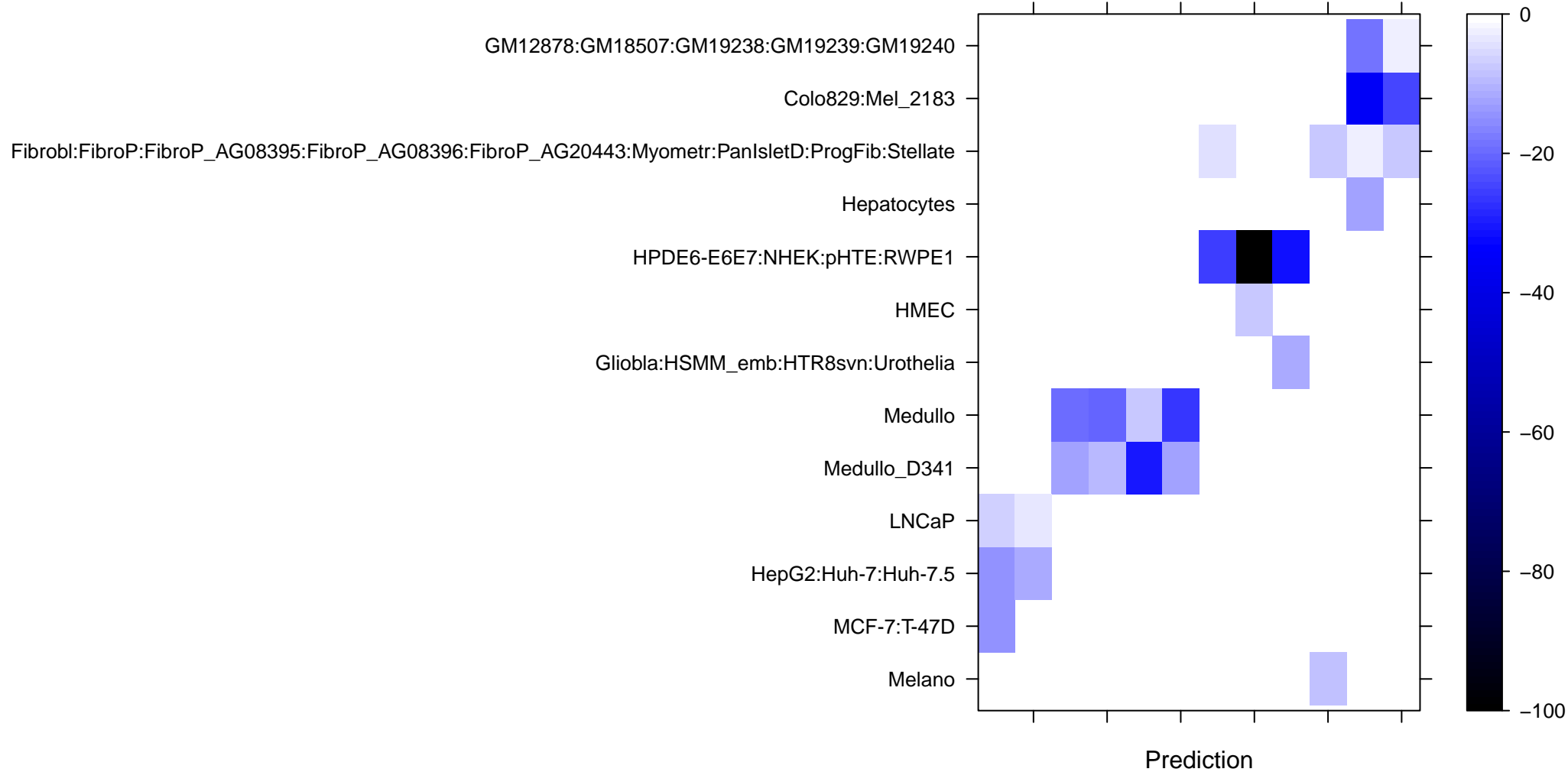

Supplement: Additional file 5: Figure S3 — Dynamic landscape of predicted TF dimers across cell types. As in Figure 5, but for motif dimers predicted in Duke DNase-seq data. [file 1471-2164-15-208-S5.pdf]

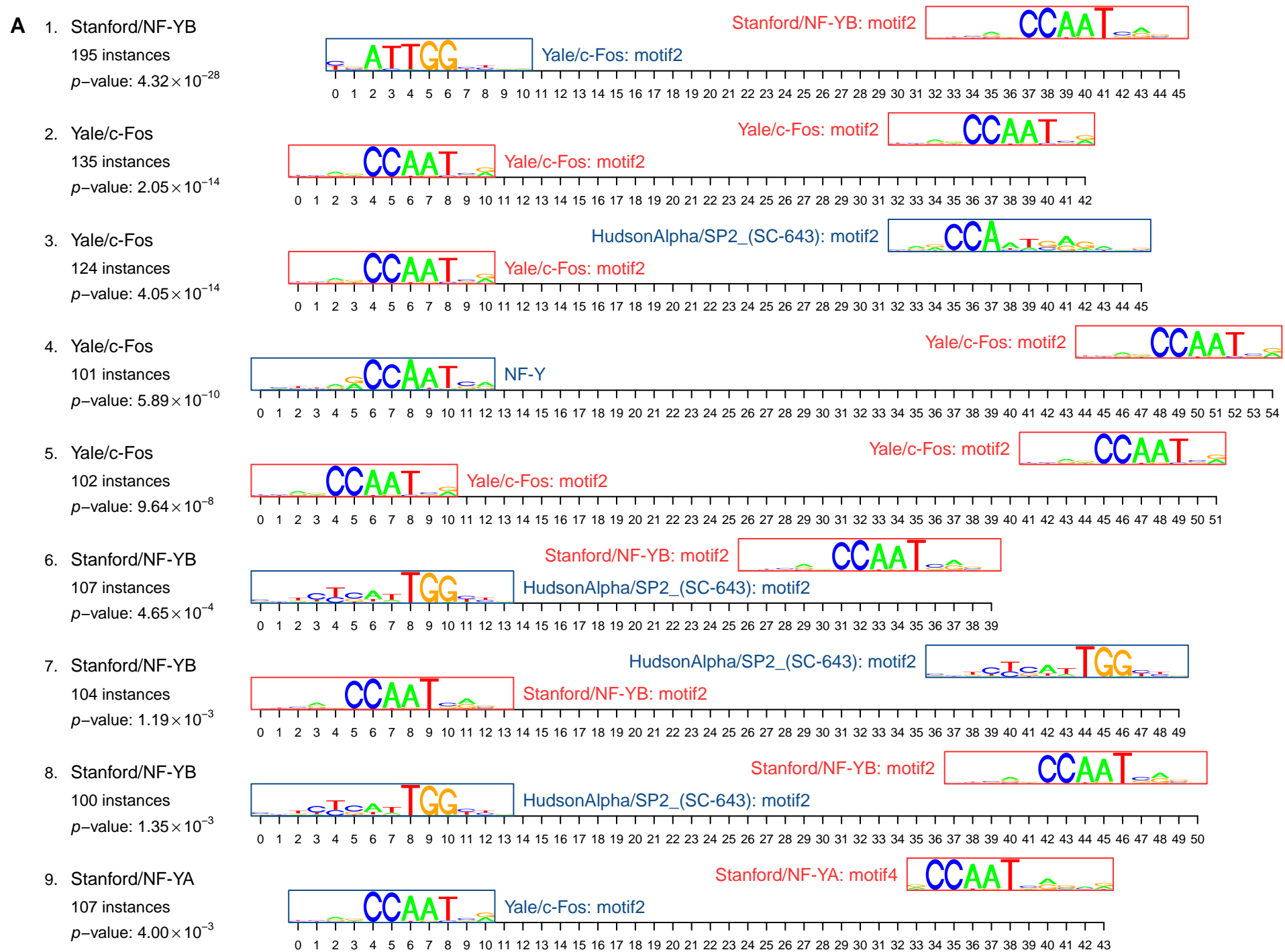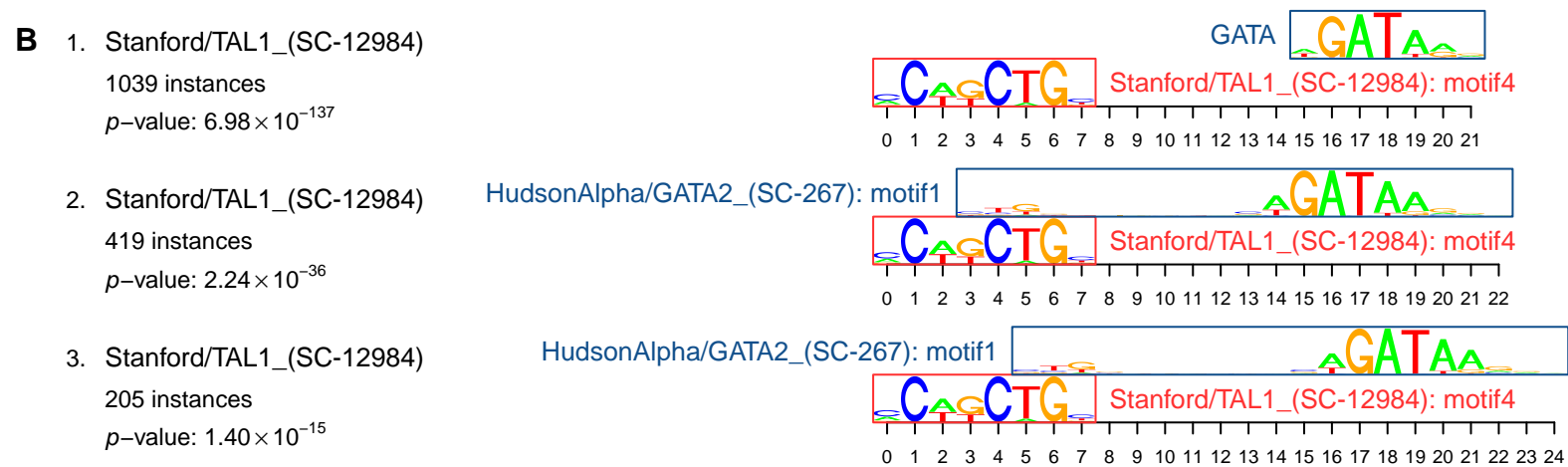

Supplement: Additional file 6: Figure S4 — Predicted long range motif dimers in K562 ChIP-seq data. As in Figure 4, (A) NF-Y homotypic dimers and (B) GATA–E-box heterodimers predicted in K562 ChIP-seq data are shown in detail. [file 1471-2164-15-208-S6.pdf]
